# Identification of the high-risk area for schistosomiasis transmission in China based on information value and machine learning: a newly data-driven modeling attempt

**DOI:** 10.1186/s40249-021-00874-9

**Published:** 2021-06-27

**Authors:** Yan-Feng Gong, Ling-Qian Zhu, Yin-Long Li, Li-Juan Zhang, Jing-Bo Xue, Shang Xia, Shan Lv, Jing Xu, Shi-Zhu Li

**Affiliations:** 1 National Institute of Parasitic Diseases, Chinese Center for Disease Control and Prevention; Chinese Center for Tropical Diseases Research; HC Key Laboratory of Parasite and Vector Biology; WHO Collaborating Centre for Tropical Diseases; National Center for International Research on Tropical Diseases, Shanghai, 200025 China; 2 School of Global Health, Chinese Center for Tropical Diseases Research, Shanghai Jiao Tong University School of Medicine, Shanghai, 200025 China

**Keywords:** Schistosomiasis, Risk prediction, Information value, Machine learning, China

## Abstract

**Background:**

Schistosomiasis control is striving forward to transmission interruption and even elimination, evidence-lead control is of vital importance to eliminate the hidden dangers of schistosomiasis. This study attempts to identify high risk areas of schistosomiasis in China by using information value and machine learning.

**Methods:**

The local case distribution from schistosomiasis surveillance data in China between 2005 and 2019 was assessed based on 19 variables including climate, geography, and social economy. Seven models were built in three categories including information value (IV), three machine learning models [logistic regression (LR), random forest (RF), generalized boosted model (GBM)], and three coupled models (IV + LR, IV + RF, IV + GBM). Accuracy, area under the curve (AUC), and F1-score were used to evaluate the prediction performance of the models. The optimal model was selected to predict the risk distribution for schistosomiasis.

**Results:**

There is a more prone to schistosomiasis epidemic provided that paddy fields, grasslands, less than 2.5 km from the waterway, annual average temperature of 11.5–19.0 °C, annual average rainfall of 1000–1550 mm. IV + GBM had the highest prediction effect (accuracy = 0.878, AUC = 0.902, F1 = 0.920) compared with the other six models. The results of IV + GBM showed that the risk areas are mainly distributed in the coastal regions of the middle and lower reaches of the Yangtze River, the Poyang Lake region, and the Dongting Lake region. High-risk areas are primarily distributed in eastern Changde, western Yueyang, northeastern Yiyang, middle Changsha of Hunan province; southern Jiujiang, northern Nanchang, northeastern Shangrao, eastern Yichun in Jiangxi province; southern Jingzhou, southern Xiantao, middle Wuhan in Hubei province; southern Anqing, northwestern Guichi, eastern Wuhu in Anhui province; middle Meishan, northern Leshan, and the middle of Liangshan in Sichuan province.

**Conclusions:**

The risk of schistosomiasis transmission in China still exists, with high-risk areas relatively concentrated in the coastal regions of the middle and lower reaches of the Yangtze River. Coupled models of IV and machine learning provide for effective analysis and prediction, forming a scientific basis for evidence-lead surveillance and control.

**Graphic Abstract:**

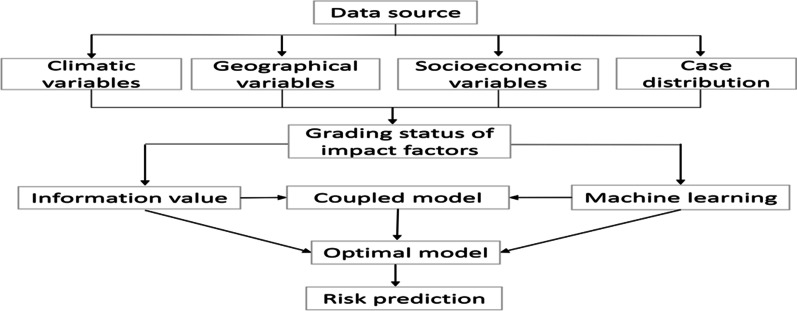

**Supplementary Information:**

The online version contains supplementary material available at 10.1186/s40249-021-00874-9.

## Background

As one of 20 neglected tropical diseases, schistosomiasis is a typical zoonotic parasitic disease that remains a major public health problem worldwide [1]. In the 1950s, schistosomiasis was endemic in 12 southern Chinese provinces in close proximity to the Yangtze River. China was one of the countries with the heaviest schistosomiasis burden with more than 10 million patients. Over the past 70 years of active control, China's schistosomiasis control program has achieved remarkable success [2]. By the end of 2020, 337 (74.9%) of the 450 schistosomiasis endemic counties in China had achieved the elimination standard, 97 (21.6%) have achieved the transmission blocking standard and 16 (3.6%) have achieved transmission control [3]. However, the risk of schistosomiasis transmission still exists in China because many natural conditions and socio-economic factors involved in the process of schistosomiasis transmission are difficult to change fundamentally in the short term [2, 3]. China’s 13th Five-Year Plan for national schistosomiasis control identifies risk monitoring and early warning to be essential to reduce potential transmission risk. Prediction model design is an effective means by which to achieve accurate monitoring and evidence-lead control of schistosomiasis [4].

There are two methods for infectious disease risk prediction: a knowledge-driven method (qualitative method), and a data-driven method (quantitative method) [5]. There are four components to the process of development: epidemic data processing, environmental factor selection, model construction, and model evaluation. In particular, the application of geographic information system (GIS), remote sensing (RS), and global positioning system (GPS) in infectious disease research accelerates the development of quantitative risk prediction [6]. Commonly used qualitative methods are the analytic hierarchy process (AHP) and the Delphi method. For example, Ajakaye et al. [7] used AHP to evaluate the transmission risk of schistosomiasis in Nigeria. Yang et al. [8] used the Delphi method to establish a schistosomiasis early warning index in the middle and lower reaches of the Yangtze River. The results for early warning were consistent with epidemic levels based on a recent epidemiological survey. A single quantitative method or a combination of multiple quantitative methods is frequently used. Solano-Villarreal et al. [9] used a boosted regression tree to study the transmission risk of malaria in the Loreto area. Xia et al. [10] combined a variety of classification algorithms including random forest (RF) and a generalized boosted model (GBM) in BioMod2, to construct a combined model that predicted the potential distribution of *Oncomelania hupensis* in the Dongting Lake region. The combined model had greater prediction accuracy.

Information value (IV) is derived by statistical quantitative analysis of data based on information theory. A model is based on the influencing factors of an epidemic as well as an evaluation of risk for the region [11]. As an example, Rai [12] used IV to establish a malaria susceptibility index. IV has high modeling efficiency and can judge the weight of various influencing factors. Classification algorithms such as logistic regression (LR), RF, and GBM can determine the weight of each influencing factor [5]. IV and classification algorithms can predict vector-borne infectious disease during the initial stage. For example, Chen et al. [13] used a coupled model of IV and LR (IV + LR) to predict hot spots of hemorrhagic fever with renal syndrome in Hunan Province of China, resulting in more accurate prediction. The application of information value combines with other models for risk assessment of infectious diseases is also increasing, which makes up for the lack of simple information value model, and simple machine learning. Based on epidemic data and related environmental factors, we used IV combined with LR, RF, and GBM respectively, to evaluate and predict the risk for schistosomiasis transmission. The purpose of this study was to compare different methods to predict the high-risk distribution of schistosomiasis, so as to provide a methodological basis for evidence-lead control of schistosomiasis.

## Methods

### Study area

The study area included 31 provinces (municipalities and autonomous regions) in the mainland of China. China is rich in geomorphic resources, with many lakes and beaches as well as a wide range of tropical and subtropical monsoon climates. Areas around lakes tend to have a gentle climate with abundant rainfall and vegetation suitable for the breeding of *O. hupensis*. This combination of factors increases the residents' risk for schistosomiasis, especially in the south of the Yangtze River Basin.

### Data collection

#### Case and non-case data

**Fig. 1:**
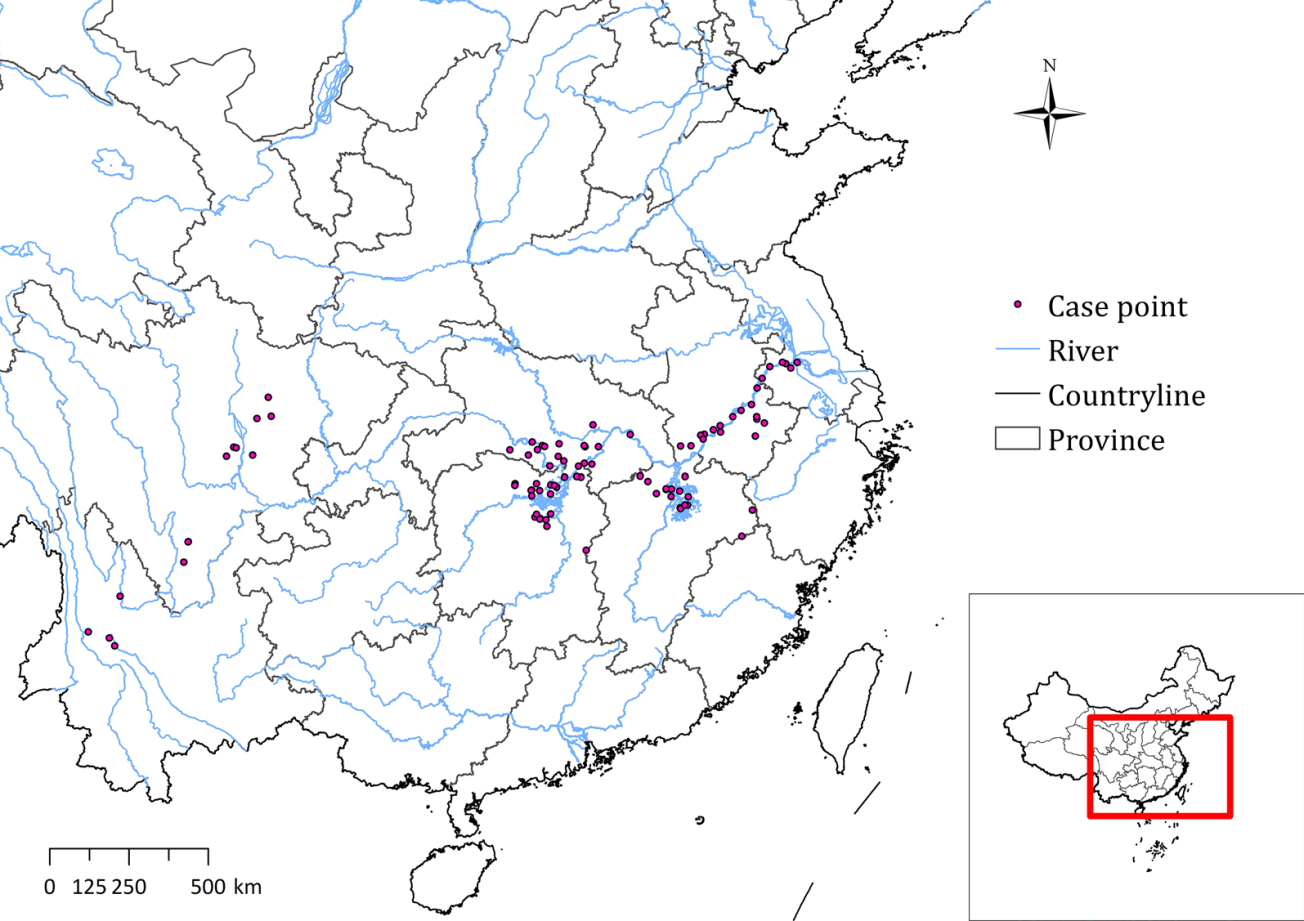
Case location and river distribution in this study

Schistosomiasis data were derived from the national schistosomiasis survey of 2005–2019 [14, 15]. Villages with indigenous cases were selected as distribution points (Fig. 1). Longitude and latitude coordinates of the distribution points were identified with the Baidu map coordinate picking system (http://api.map.baidu.com/lbsapi/getpoint/index.html). The model calibration required both case and non-case data, but non-occurrence point were usually ignored and not recorded in the field survey. This study randomly selected coordinate points for nonexistent points in non-endemic counties adjacent to schistosomiasis endemic counties based on a ratio of 1:2 in order to increase the discrimination of environmental factors.

**Fig. 2:**
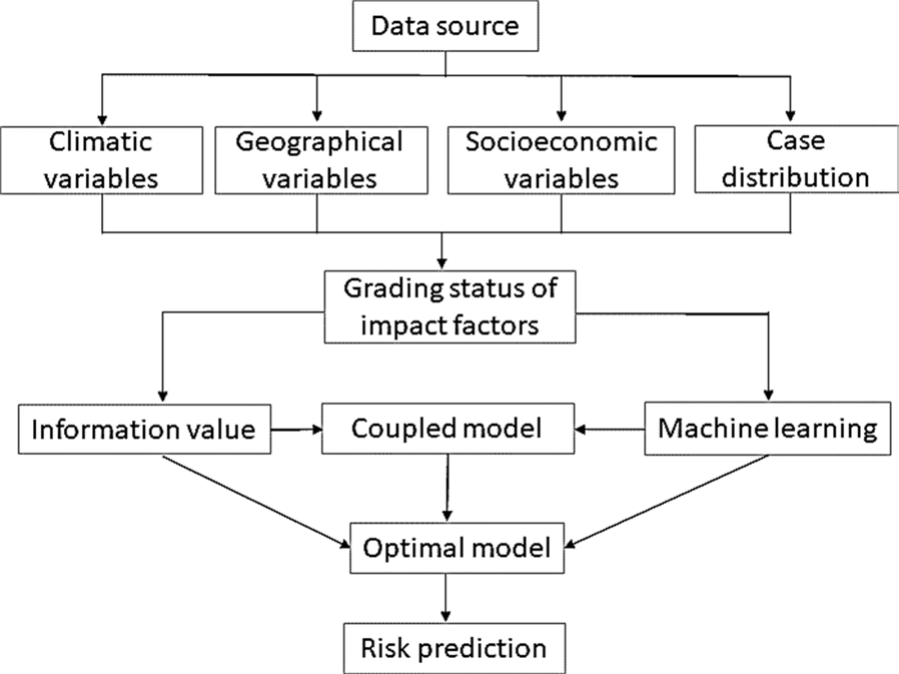
Implementation path of model building

#### Environmental data

Environmental variables related to schistosomiasis and its vector snail distribution were collected. This included ten climate variables, six geographical variables, and three socio-economic variables, as shown in Table 1. Among the climate related variables, four types of background meteorological data were derived from the Resource and Environmental Science and Data Center of the Chinese Academy of Sciences (http://www.resdc.cn/) and represent conventional climate conditions. The other six bioclimatic variables were based on the high-resolution climate data website WorldClim (https://www.worldclim.org/). Those data included mean diurnal temperature range (BIO2), temperature annual range (BIO7), mean temperature of the warmest quarter (BIO10), mean temperature of the coldest quarter (BIO11), precipitation of the wettest quarter (BIO16), and precipitation of driest quarter (BIO17). These data represent extreme climatic conditions and limit the distribution range of *S. japonicum* and *O. hupensis.* Elevation and annual normalized vegetation index for the geographic environmental variables were from the Resource and Environment Science and Data Center of Chinese Academy of Sciences (http://www.resdc.cn/). Landform types and land use types are from the National Earth System Science Data Sharing Platform (http://www.geodata.cn). Distance to waterways was obtained from WorldPop (https://www.worldpop.org/). Socio-economic variables including gross domestic product (GDP), population density, and night light, which were obtained from the Resource and Environment Science and Data Center of Chinese Academy of Sciences (http://www.resdc.cn/). ArcGIS 10.2 (Environmental Systems Research Institute, Inc, USA) was used to trim all environmental variables to the same spatial range and then resampled to a spatial resolution of 1 km × 1 km.

**Table 1:** Summary of environmental variables related to the distribution of schistosomiasis and *Oncomelania hupensis*

Category	Variable name	Definition	Source
Climate variables	AR	Aridity	http://www.resdc.cn/
	IM	Index of moisture	
	AAP	Average annual precipitation	
	AAT	Average annual temperature	
	BIO2	Mean diurnal temperature range	https://www.worldclim.org/
	BIO7	Temperature annual range	
	BIO10	Mean temperature of warmest quarter	
	BIO11	Mean temperature of coldest quarter	
	BIO16	Precipitation of wettest quarter	
	BIO17	Precipitation of driest quarter	
Geographic variables	LF	Landform	http://www.geodate.cn/
	LD	Land use	
	SLOPE	Slope	https://www.worldpop.org/
	DST	Distance to waterway	
	EL	Elevation	http://www.resdc.cn/
	ANDVI	Annual normalized difference vegetation index	
Socio -economic variables	GDP	Gross domestic product	http://www.resdc.cn/
	DP	Density of population	
	NTL	Night-time lights	

### Analytical modeling

#### Information value (IV) model

IV [13] uses the frequency or density of schistosomiasis occurrence to reflect the risk effect of different influencing factors and their sub-intervals. An IV is calculated that represents the contribution of different influencing factors related to the occurrence of schistosomiasis. A regional risk assessment for schistosomiasis transmission is realized through the spatial superposition of multi-factor information [13]. The formula is as follows:

$$I = \sum\nolimits_{{i = 1}}^{n} {lg\frac{{Ni/N}}{{Si/S}}}$$

where *n* is the total number of evaluation factors selected in the study area; *Ni* is the number of schistosomiasis units distributed in evaluation factors; *N* is the total number of schistosomiasis units in the region; *Si* is the number of units with evaluation factors in the region; *S* is the total number of evaluation units in the region.

When *I* is positive, the combination of multiple factors will increase the risk of schistosomiasis in grid cells, otherwise, it is not conducive to the occurrence of schistosomiasis. The IV model was implemented in R 4.0.0 (R Development Core Team; R foundation for Statistical Computing; Vienna, Austria) using the "scorecard" package (Table 2).

**Table 2:** Confusion matrix of binary classification results

Predicted result	Predicted presence	Predicted absence
Investigated presence	a	b
Investigated absence	c	d

a. True predicted presence; b. False predicted presence; c. False predicted absence; d. True predicted absence

#### Machine learning

A logistic regression model (LR) [16] is a statistical nonlinear classification method based on logit transformation, which is widely used in classification and prediction tasks due to its simplicity, rapidity, and relative accuracy. A random forest model (RF) [17] is a predictive model based on statistical analysis principles formed by the combination of multiple decision trees. A generalized boosted model or gradient boosting machine (GBM) [18] is based on two algorithms: regression trees and gradient boosting. It builds multiple regression trees on the basis of self-learning and multiple random selections. The machine learning models associate the epidemic data with the drivers, and then apply the association to the study area to estimate the disease risk of schistosomiasis. LR and GBM uses the "H20" package, and RF uses the "randomForest" package to implement the modeling process in R 4.0.0.

#### Model coupling

Using calculated information value “*I*” to replace the corresponding frequency ratio of LR, sample variable values for RF and GBM, and coupled models (IV + LR, IV + RF, and IV + GBM) are obtained. The modeling path of this research is shown in Fig. 2.

### Model evaluation

The sample data were randomly divided into two parts: 75% as training samples for model construction, and 25% as test samples to evaluate the accuracy, referred to relevant literature [19]. A confusion matrix was used to reflect the comprehensive performance of the models (Table 2). The accuracy, area under the curve (AUC), and F1-score derived from the confusion matrix were used to evaluate the prediction effect comprehensively.

Accuracy = (a + d)/(a + b + c + d); F1 = (2(a/(a + b) × a/(a + c))/(a/(a + b) + a/(a + c)). The higher the accuracy and F1, the better the prediction effect of the model [20]. The AUC is derived from the receiver operating characteristic curve, which takes the true positive rate (a/a + c) as the ordinate and the false positive rate (b/b + d) as the abscissa according to a series of different dichotomies. The AUC threshold is (0, 1), the larger the AUC value, the better the performance of the model [21].

### Risk visualization analysis

We selected the optimal model based on the evaluation indicator and calculated the transmission risk index for the study area. Then, the area was divided into four levels: no-risk area (0.00–0.40), low-risk area (0.41–0.60), medium-risk area (0.61–0.80), and high-risk area (0.81–1.00) [22].

## Results

### Correlation analysis among schistosomiasis and environmental factors

Based on the principle of chi-square binning, the upper limit of binning is set to 8, and the IV of different levels of influencing factors is calculated according to the binning situation (Table 3). When annual average temperature is 11.5–19.0 °C, the annual average rainfall is 1000–1550 mm, the dryness is 66–92%, and the wetness index is 45–70%, schistosomiasis is more likely to occur. In this geographic environment, the risk of schistosomiasis transmission is higher when the distance from waterways is less than 2.5 km, the altitude is less than 100 m, the land use is paddy field, grassland, and water area, and the landform type is plain. Extreme climate and geographic conditions are not conducive to the spread of schistosomiasis: for example, annual rainfall of less than 1000 mm or more than 1550 mm, annual average temperature of less than 11.5 °C or more than 19 °C, average temperature during the hottest season of less than 27 °C, rainfall in the wettest season of less than 500 mm, and distance to the waterway of more than 3 km, with a slope greater than six (Table 4).

**Table 3:** Number and meaning of environmental factor classification based on the principle of chi-square binning

Factors	Number	Classification index
AAP (mm)	8	< 850; 850–950; 950–1000; 1000–1350; 1350–1450; 1450–1500; 1500–1550; > 1550
AAT (°C)	8	< 11.5; 11.5–16.0; 16.0–17.0; 17.0–17.5; 17.5–18.0; 18.0–18.5; 18.5–19.0; > 19.0
IM (%)	8	< 45; 45–50; 50–55; 55–60; 60–65; 65–70; 70–90; > 90
AR (%)	8	< 62; 62–66; 66–68; 68–72; 72–74; 74–92; 92–95; > 95
BIO2	8	< 7.3; 7.3–7.8; 7.8–7.9; 7.9–8.2; 8.2–8.6; 8.6–9.3; 9.3–9.9; > 9.9
BIO7	8	< 24; 24–27.5; 27.5–29; 29–31; 31–31.5; 31.5–33; 33–33.5; > 33.5
BIO10 (°C)	8	< 17; 17–20; 20–22; 22–25; 25–26.5; 26.5–27; 27–28; > 28
BIO11 (°C)	8	< 5.8; 5.8–6.0; 6.0–6.2; 6.2–6.4; 6.4–6.6; 6.6–7.6; 7.6–8.6; > 8.6
BIO16 (mm)	8	< 440; 440–460; 460–480; 480–500; 500–520; 520–540; 540–560; > 560
BIO17 (mm)	8	< 20; 20–50; 50–130; 130–140; 140–155; 155–160; 160–175; > 175
LF	6	Plains; terraces; hills; small undulating mountains; medium undulating mountains; large undulating mountains
LD	7	Paddy field; dry land; woodland; grassland; water area; urban and rural residential land; unused land
EL (m)	7	< 50; 50–100; 100–450; 450–700; 700–2150; 2150–2500; > 2500
SLOPE (°)	8	< 2; 2–3; 3–6; 6–9; 9–13; 13–22; 22–29; > 29
DST (km)	8	< 0.5; 0.5–1.0; 1.0–1.5; 1.5–2; 2–2.5; 2.5–3; 3–3.5; > 3.5
ANDVI	8	< 0.78; 0.78–0.79; 0.79–0.8; 0.8–0.81; 0.81–0.82; 0.82–0.83; 0.83–0.84; > 0.84
GDP (10 000/km^2^)	7	< 50; 50–100; 100–150; 150–250; 250–350; 350–800; 800–1000; > 1000
DP (Person/km^2^)	8	< 100; 100–150; 150–200; 200–250; 250–400; 400–450; 450–550; > 550
NTL	8	< 0.08; 0.08–0.10; 0.10–0.12; 0.12–0.14; 0.14–0.16; 0.16–0.18; 0.18–0.54; > 0.54

*AAP* average annual temperature, *AAT* annual accumulated temperature, *IM* index of moisture, *AR* aridity, *BIO2* mean diurnal temperature range, *BIO7* temperature annual range, *BIO10* mean temperature of warmest quarter, *BIO11* mean temperature of coldest quarter, *BIO16* mean precipitation of wettest quarter, *BIO17* mean precipitation of driest quarter, *LF* landform, *LD* land use, *SLOPE* slope, *DST* distance to waterway, *EL* elevation, *ANDVI* annual normalized difference vegetation index, *GDP* gross domestic product, *DP* density of population, *NTL* night-time lights

**Table 4:** Results for grading information value by environmental influencing factors

Grade	1	2	3	4	5	6	7	8
AAP	− 1.435	− 0.941	− 0.789	0.223	0.901	1.219	0.118	− 0.811
AAT	− 2.970	0.411	0.647	0.693	0.544	1.067	0.582	− 0.305
IM	− 0.498	0.916	0.095	0.693	0.818	1.587	− 0.288	− 1.466
AR	− 1.224	− 0.693	0.836	0.773	0.383	0.228	− 0.801	− 1.447
BIO2	0.547	1.176	0.319	0.323	0	− 0.553	− 1.194	− 1.269
BIO7	− 0.406	− 0.651	− 1.504	− 1.355	0.357	0.774	0.568	− 1.082
BIO10	− 2.773	− 0.838	− 0.693	− 1.674	− 0.827	− 1.584	0.894	1.192
BIO11	− 1.064	1.121	1.121	1.118	0.847	0.228	− 0.773	− 0.406
BIO16	− 0.916	− 0.074	− 0.442	− 1.065	0.598	0.223	0.811	0.180
BIO17	− 2.110	− 0.887	− 0.203	0.499	1.421	0.767	0.534	− 0.095
LF	0.950	1.068	0.766	− 0.300	− 0.742	− 1.789		
LD	0.347	0.169	− 0.266	0.342	0.234	0.123	− 1.634	
SLOPE	0.841	0	− 0.187	− 1.099	− 2.485	− 0.821	− 0.949	− 1.946
DST	0.395	0.560	0.821	0.442	0.147	− 0.406	0	− 0.515
EL	0.959	0.195	− 1.126	− 0.167	− 0.975	− 0.651	− 2.169	
ANDVI	0.227	− 0.105	− 0.486	0.223	− 0.452	− 0.223	− 1.299	− 0.256
GDP	− 1.052	− 0.065	0.035	0.773	0.619	0.218	0.211	0.511
DP	− 0.946	− 0.102	− 1.179	0.560	0.431	0.368	1.099	0.621
NTL	− 0.887	− 0.674	0.111	− 0.827	0.143	0.470	0.186	0.450

*AAP* average annual temperature, *AAT* annual accumulated temperature, *IM* index of moisture, *AR* aridity, *BIO2* mean diurnal temperature range, *BIO7* mean temperature annual range, *BIO10* mean temperature of warmest quarter, *BIO11* mean temperature of coldest quarter; *BIO16* mean precipitation of wettest quarter, *BIO17* mean precipitation of driest quarter, *LF* landform, *LD* land use, *SLOPE* slope, *DST* distance to waterway, *EL* elevation, *ANDVI* annual normalized difference vegetation index, *GDP* gross domestic product, *DP* density of population, *NTL* night-time lights

### Comparison of prediction results based on the seven models

Prediction results for IV, by three machine learning models (LR, RF, GBM), and three coupled models (IV + LR, IV + RF, IV + GBM) are shown in Additional file 1: Fig. 1, Additional file 2: Fig. 2, Additional file 3: Fig. 3, Additional file 4: Fig. 4, Additional file 5: Fig. 5, Additional file 6: Fig. 6, Additional file 7: Fig. 7. IV shows that the schistosomiasis risk is widely distributed throughout the Yangtze River Basin and its southern areas. High-risk areas are mainly distributed in southern Hubei, northern Hunan, northwestern Jiangxi, and central Anhui. Prediction results for the three machine learning models had similarities and differences. The possibility for schistosomiasis transmission was mainly concentrated in the middle and lower reaches of the Yangtze River by three machine learning models. LR indicated the risk was also distributed in northern Xinjiang and southwestern Tibet. RF showed a lower risk in southern Guangzhou. GBM showed a lower risk in northern Xinjiang. Prediction results for the three coupled models were better than those for the single models. There was no obvious abnormal risk in north of the Yangtze River, although small detail differences in risk areas were observed. For example, IV + RF showed no obvious risk area in central Sichuan or northwestern Yunnan, as opposed to IV + GBM.

The predicted performance for schistosomiasis by the seven models as judged by transmission risk, accuracy, AUC, and F1 for each model was calculated (Table 5). Sorted model prediction results were ordered as follows: AUC, IV + GBM > IV + RF > GBM > IV + LR > IV > RF > LR. Overall, the coupled models had the best results, followed by the three machine models, and then the information model. The best of the three machine learning models was GBM, and the best of the three coupled models was IV + GBM (accuracy = 0.878, AUC = 0.902, F1 = 0.920).

**Table 5:** Predictive performance indicators for the seven models

Model	IV	LR	IV + LR	RF	RF + IV	GBM	IV + GBM
Accuracy	0.732	0.790	0.815	0.785	0.820	0.849	0.878
AUC	0.750	0.827	0.853	0.840	0.872	0.859	0.902
F1	0.705	0.867	0.871	0.854	0.875	0.903	0.920

*IV* information value, *LR* logistic regression, *RF* random forest, *GBM* generalized boosted model, *AUC* area under the curve

### Risk prediction of schistosomiasis transmission in China based on the optimal coupled model

Prediction results for GBM + IV showed the risk of schistosomiasis in China to be scattered through a large spatial range, although clusters appeared in southeastern Hubei province, northeastern Hunan province, northern Jiangxi province, central Anhui province, central Sichuan province, northwestern Yunnan province, and southern Jiangsu province. Superimposed on the national river map, risk areas were concentrated in the coastal areas of the middle and lower reaches of the Yangtze River, Poyang Lake region, and Dongting Lake region.

**Fig. 3:**
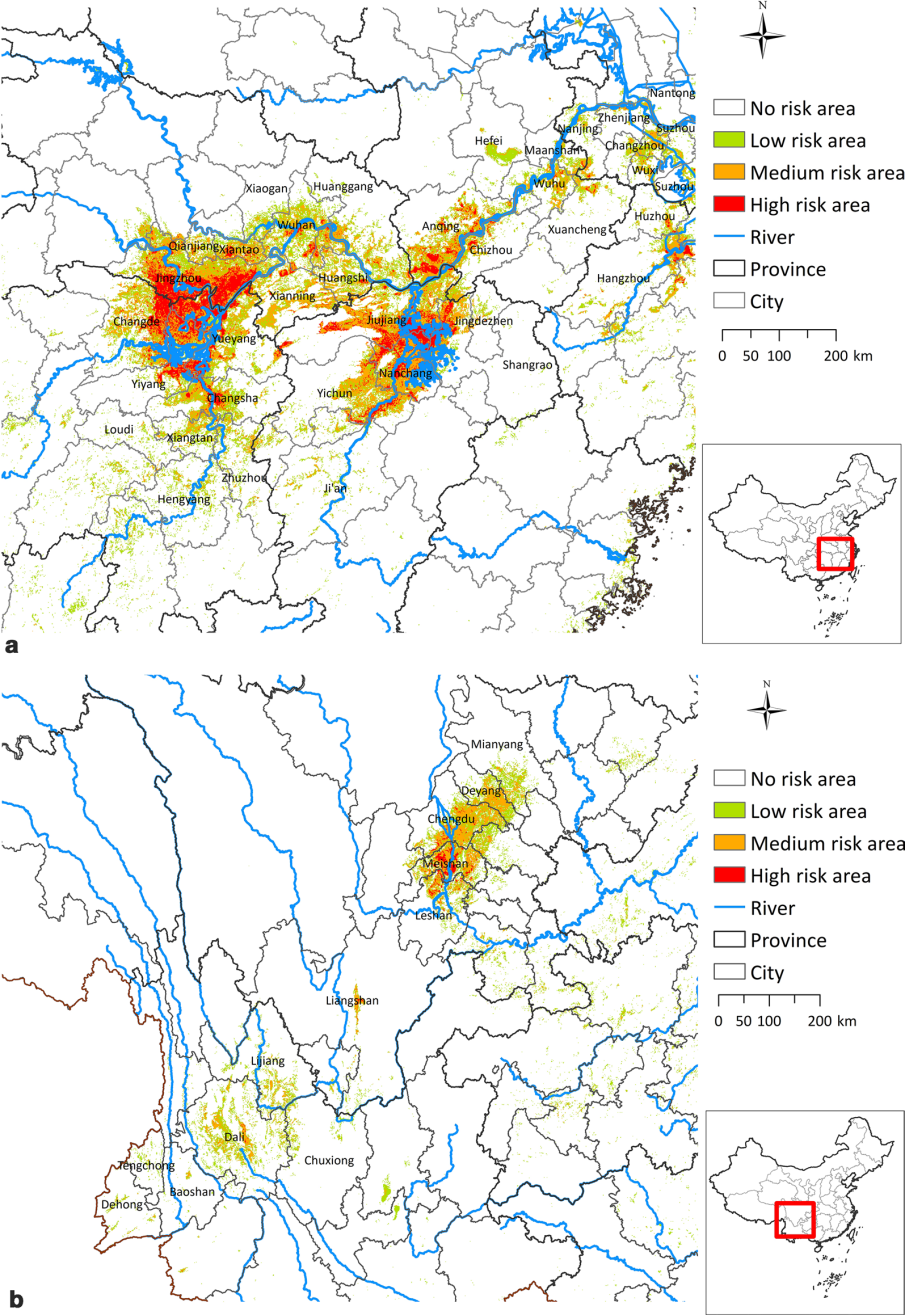
Current risk prediction for schistosomiasis in China based on the optimal coupled model

Classification of transmission risk shows that 4.7% of China is in an at-risk area and 95.3% is not. Risk areas can be divided into low-risk (2.5%), medium-risk (1.4%), and high-risk areas (0.8%). High-risk areas are primarily distributed in eastern Changde, western Yueyang, northeastern Yiyang, middle Changsha of Hunan province; southern Jiujiang, northern Nanchang, northeastern Shangrao, eastern Yichun in Jiangxi province; southern Jingzhou, southern Xiantao, middle Wuhan in Hubei province; southern Anqing, northwestern Guichi, eastern Wuhu in Anhui province; middle Meishan, northern Leshan, and the middle of Liangshan in Sichuan province (Fig. 3). Medium-risk areas and low-risk areas are distributed in areas adjacent to high-risk areas, as well as southern Jiangsu and northwestern Yunnan.

## Discussion

Due to the unique life history of *S. japonicum* and *O. hupensis*, as well as the numerous terminal hosts of *S. japonicum*, the epidemic process for schistosomiasis is exceedingly complex. Geographic, climatic, socio-economic, and other factors affect the scope and degree of schistosomiasis [23]. In this study, coupled models for IV and machine learning were used to evaluate factors that interfere with schistosomiasis transmission. A spatial distribution pattern of potential risks provided a support tool for the formulation of macroscopic schistosomiasis control strategies and the development of a quantitative risk assessment model for communicable diseases.

In our study, coupled models of IV and machine learning were applied to schistosomiasis transmission risk. Coupled models were used to establish statistical relationships among case distribution and environmental factors, providing a new method for analysis and prediction of hot spots of schistosomiasis transmission. By comparing the seven model indicators, we found that coupled models have better prediction accuracy than IV and machine learning models alone. The prediction results more accurately reflected the spatial distribution of risk for schistosomiasis. Differences in prediction results and goodness of fit were found for the seven models, reflecting model uncertainty. A final, optimal model, GBM + IV, was selected to predict the risk for schistosomiasis transmission. That model reduced the errors associated with the other models. Machine learning algorithms cannot express the relationships among the influencing factor’s internal levels and the occurrence of schistosomiasis. IV does not consider differences in the weight contribution of influencing factors [24]. The higher success rate for the coupled model is that it considers the internal level of influencing factors and the weight of different influencing factors in relationship to schistosomiasis [25]. Therefore, risk prediction results are more scientific and reasonable.

Predicted middle-risk and high-risk areas based on the optimal coupled model were consistent with the areas of schistosomiasis transmission control and blocking in China [26]. Combined with the distribution of water areas in China, the coastal areas of middle and lower reaches of the Yangtze River, the Poyang Lake region, and the Dongting Lake region are the high-risk areas for schistosomiasis spread. This is likely due to the wide distribution and high density of *O. hupensis* in those areas [27]. Further, there are numerous water conservancy projects, frequent population flow, developed animal husbandry industries, and increased opportunities for human and animal contact, placing these regions at risk for schistosomiasis rebound [28, 29]. With the implementation of comprehensive control strategy focused on the control of infectious source, the distribution pattern of intermediate host, the composition and distribution trend of infectious source, and the mode of population activity in epidemic area have changed significantly. Moreover, flood disaster [30], wetland construction [31] may lead to increased risk of snail diffusion, global warming [32] will prolong the growth season of *Schistosoma* and *O. hupensis* and speed up their growth. Hence, there is a greater risk for infection in the areas described above. In the epidemic risk areas, we recommend *O. hupensis* monitoring, strengthened infection control of domestic and wild animals, and timely assessment of epidemic schistosomiasis. In this manner, the goal of schistosomiasis elimination by 2030 will be achieved [33].

The relationships among the spatial change of schistosomiasis risk and environmental factors can be explained by a biological knowledge of *S. japonicum* and snails [34]. Suitable climatic conditions, small slopes, and proximity to rivers are conducive to the growth and reproduction of *S. japonicum* and snails [35], which in turn leads to the prevalence of schistosomiasis. This study demonstrates that temperature, rainfall, altitude, and the risk of schistosomiasis transmission are closely related. Abnormal climatic conditions will have a negative impact on an epidemic, which confirms previous studies using different methods [36]. Certainly, environmental factors determine the transmission dynamics of schistosomiasis. Previous studies [37] have shown that land use greatly affects the distribution and density of snails in rice fields. When water is high and in proximity to a river, there is an increased risk for infection. This may be due to the increased risk of swimming, fishing, and agricultural activities in contact with water bodies containing cercariae [38]. This study did not find a high risk for schistosomiasis transmission in economically backward areas, which may be due to the large scope of the study. Schistosomiasis is mainly prevalent in rural villages in the middle and lower reaches of the Yangtze River. Although these villages belong to economically backward areas, their economic development level is relatively better compared with remote western areas such as villages in Xinjiang, Tibet and Gansu that does not have the natural conditions for schistosomiasis epidemic. Further, results were based on surveillance data from 2005 to 2019 in China, which is accurate and reliable. However, there may be errors in the analysis of relationships among influencing factors and transmission risk due to insufficient case numbers.

This study has some limitations. First, although IV + GBM provided high goodness of fit, the potential risk for schistosomiasis remains uncertain, because of other associated factors such as snail control, cattle grazing, water conservancy construction, and behaviors [39–41]. Second, risk prediction based on IV + GBM identified sporadic high risk in northern Zhejiang, which is inconsistent with the known elimination of schistosomiasis in Zhejiang. The reason may be that the environment in the area is very similar to that of the case distribution point, but due to the intervention of human factors, there is no longer an epidemic of schistosomiasis in Zhejiang. For the future, more variables related to disease transmission should be collected, which would enrich the data set. Further, IV combined with more classification algorithms would improve assessment. These approaches would result in better predictive model performance and provide guidance for monitoring and early warning of disease in key areas.

## Conclusions

This study confirmed that a model that combines IV and machine learning is better than a single model. Among the models, the optimal coupling model had a better predictive performance for schistosomiasis risk assessment, roughly consistent with the actual situation. These results can guide monitoring and control of schistosomiasis and serve as a reference for predicting the risk of other vector-mediated infectious diseases.

## Supplementary Information

**Additional file 1: Figure 1.** Current risk prediction for schistosomiasis inChina based on the IV model.

**Additional file 2: Figure 2.** Current risk prediction for schistosomiasis inChina based on the LR model.

**Additional file 3: Figure 3.** Current risk prediction for schistosomiasis inChina based on the RF model.

**Additional file 4: Figure 4.** Current risk prediction for schistosomiasis in China based on the GBM model.

**Additional file 5: Figure 5.** Current risk prediction for schistosomiasis in China based on the IV + LR model.

**Additional file 6: Figure 6.** Current risk prediction for schistosomiasis inChina based on the IV + RF model.

**Additional file 7: Figure 7.** Current risk prediction for schistosomiasis inChina based on the IV + GBM model.

## Data Availability

The datasets used and/or analyzed during the current study are available from the corresponding author on reasonable request.

## Abbreviations

IV: Information value
LR: Logistic regression
RF: Random forest
GBM: Generalized boosted model
AUC: Area under the curve
GIS: Geographic information system
RS: Remote sensing
GPS: Global positioning system
AHP: Analytic hierarchy process
