# Supplementary figures and images for: Identification of the high-risk area for schistosomiasis transmission in China based on information value and machine learning: a newly data-driven modeling attempt
Source: Infect Dis Poverty. 2021 Jun 27;10:88. doi: 10.1186/s40249-021-00874-9 (PMC8237418; doi:10.1186/s40249-021-00874-9)

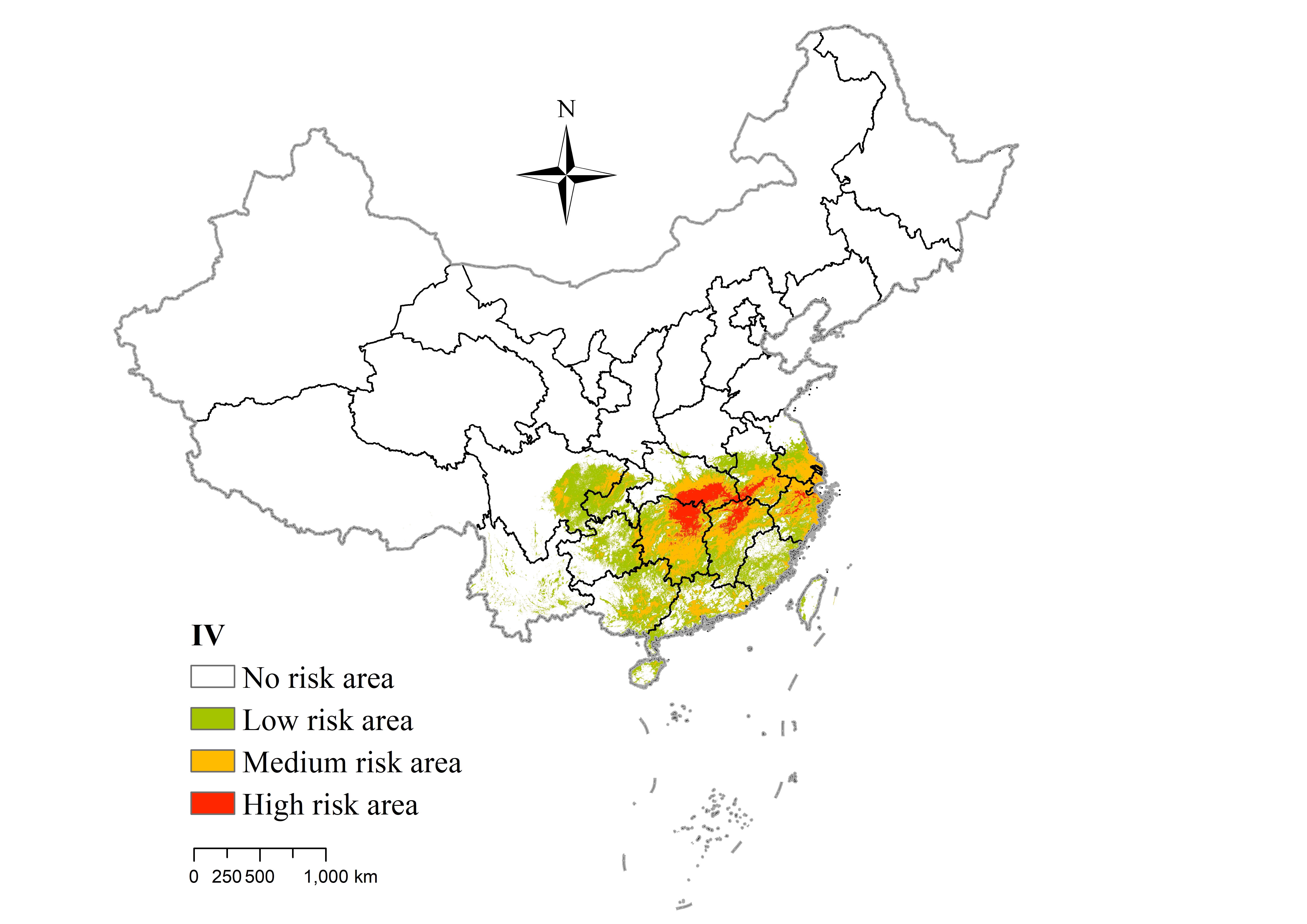

Supplement: Supplementary file 1 — Additional file 1: Figure 1. Current risk prediction for schistosomiasis inChina based on the IV model. [file 40249_2021_874_MOESM1_ESM.jpg]

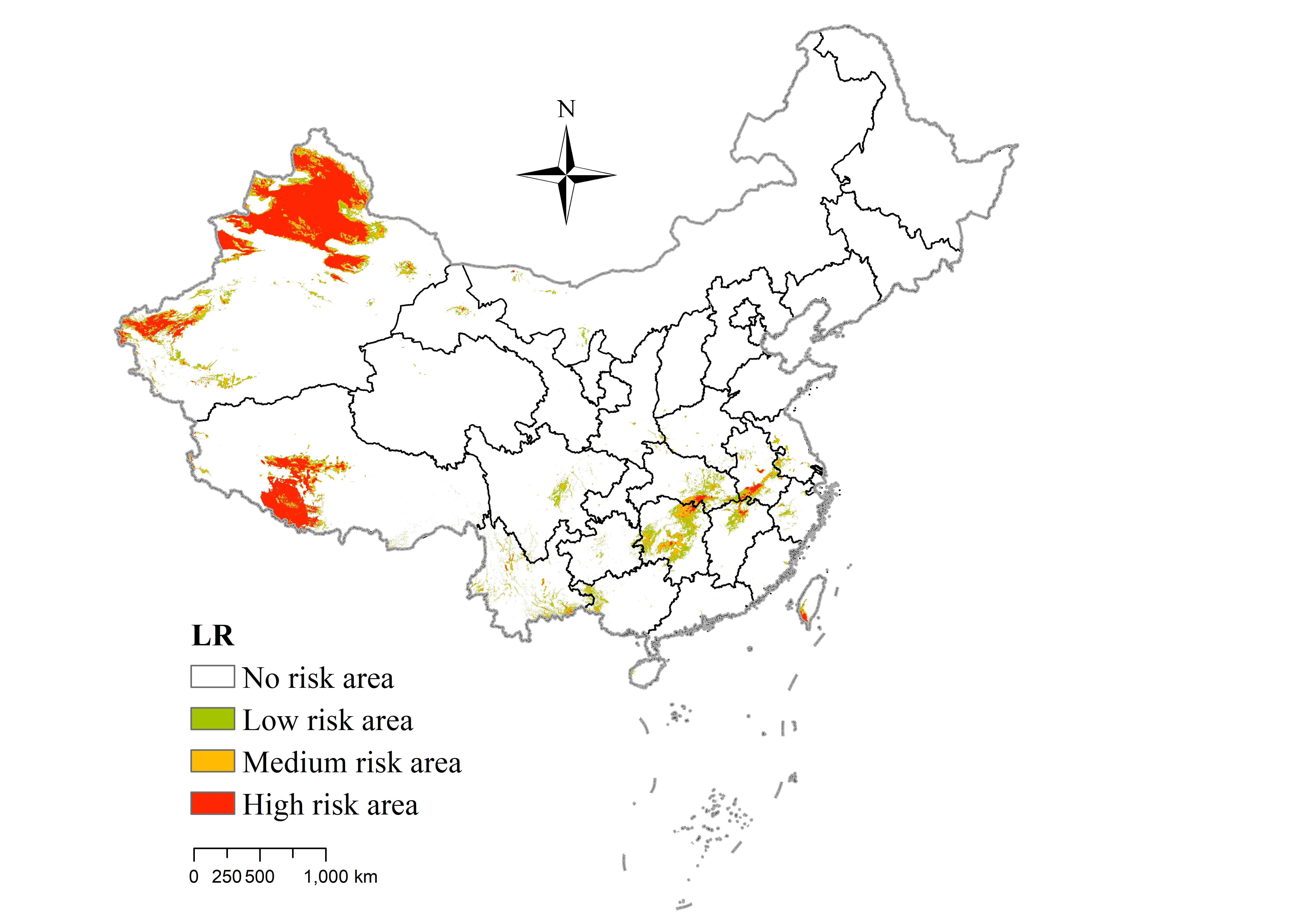

Supplement: Supplementary file 2 — Additional file 2: Figure 2. Current risk prediction for schistosomiasis inChina based on the LR model. [file 40249_2021_874_MOESM2_ESM.jpg]

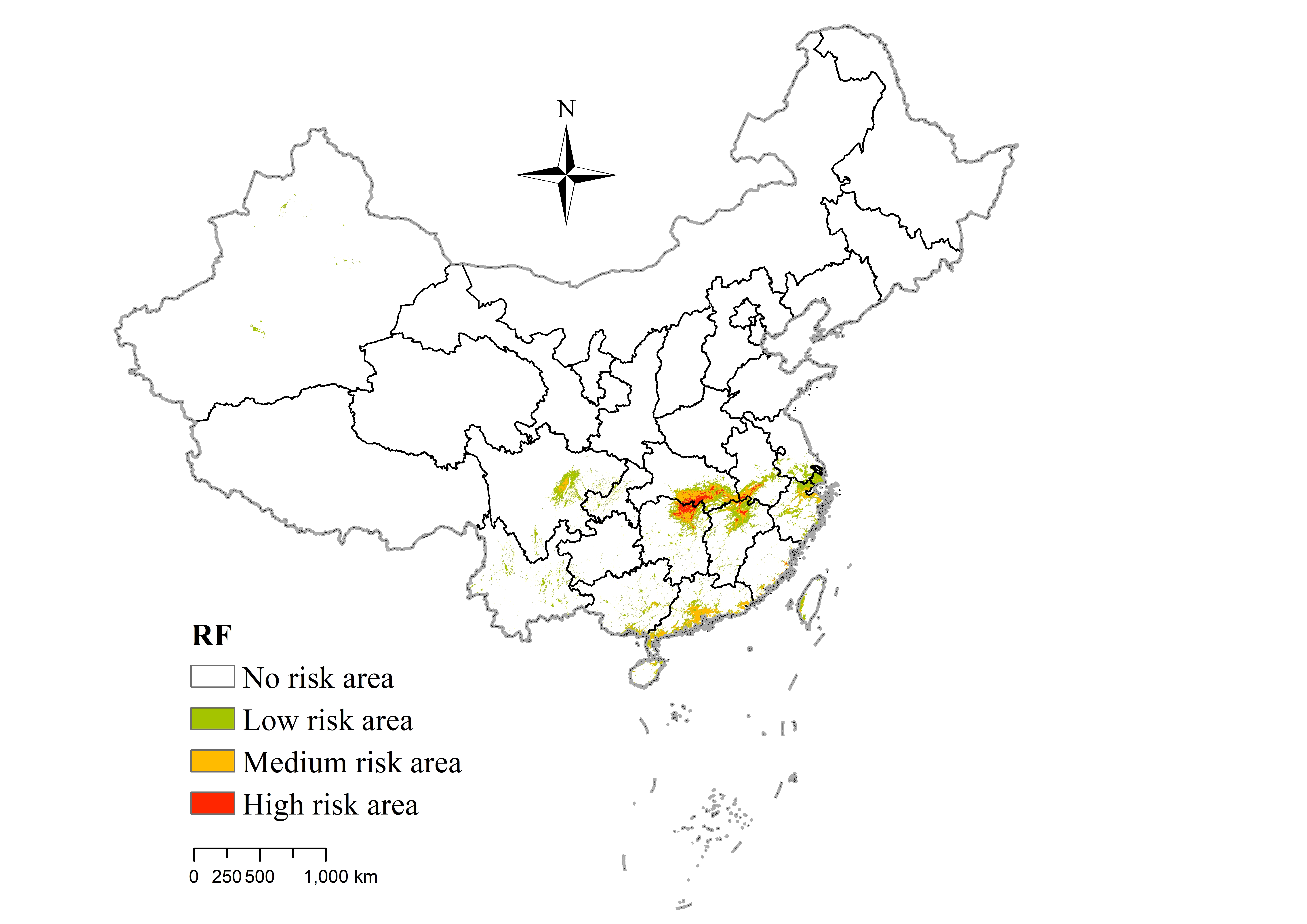

Supplement: Supplementary file 3 — Additional file 3: Figure 3. Current risk prediction for schistosomiasis inChina based on the RF model. [file 40249_2021_874_MOESM3_ESM.jpg]

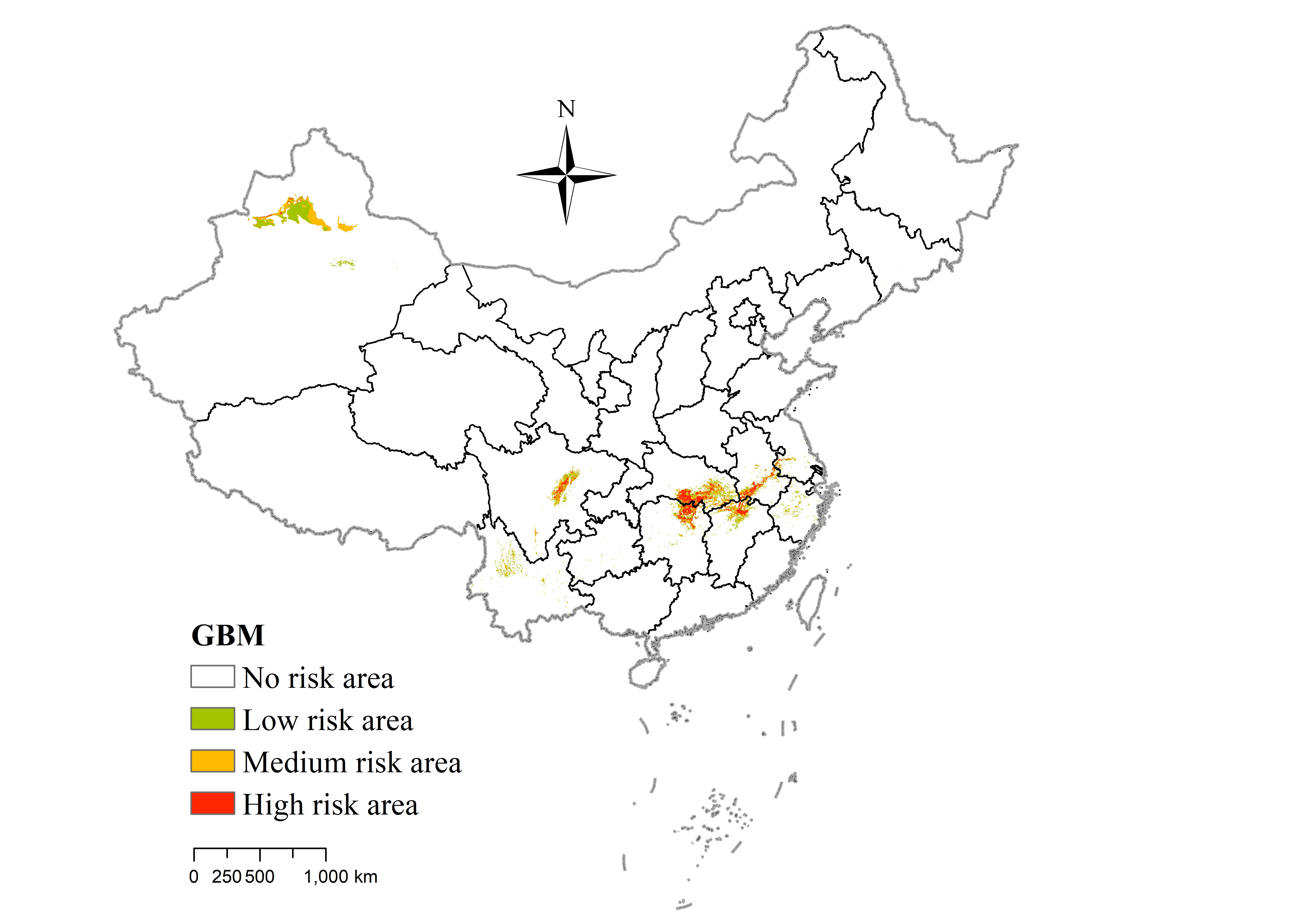

Supplement: Supplementary file 4 — Additional file 4: Figure 4. Current risk prediction for schistosomiasis in China based on the GBM model. [file 40249_2021_874_MOESM4_ESM.jpg]

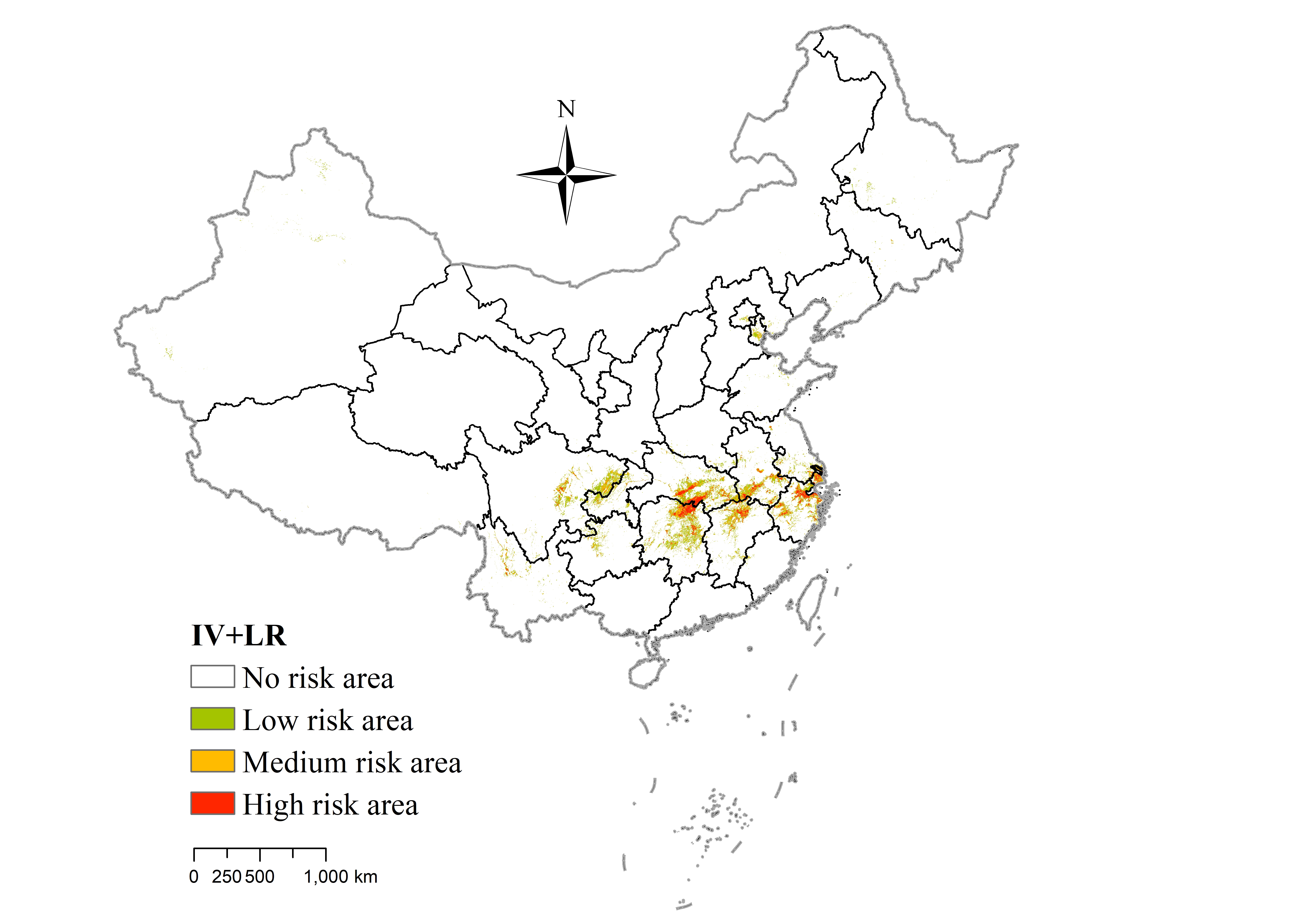

Supplement: Supplementary file 5 — Additional file 5: Figure 5. Current risk prediction for schistosomiasis in China based on the IV + LR model. [file 40249_2021_874_MOESM5_ESM.jpg]

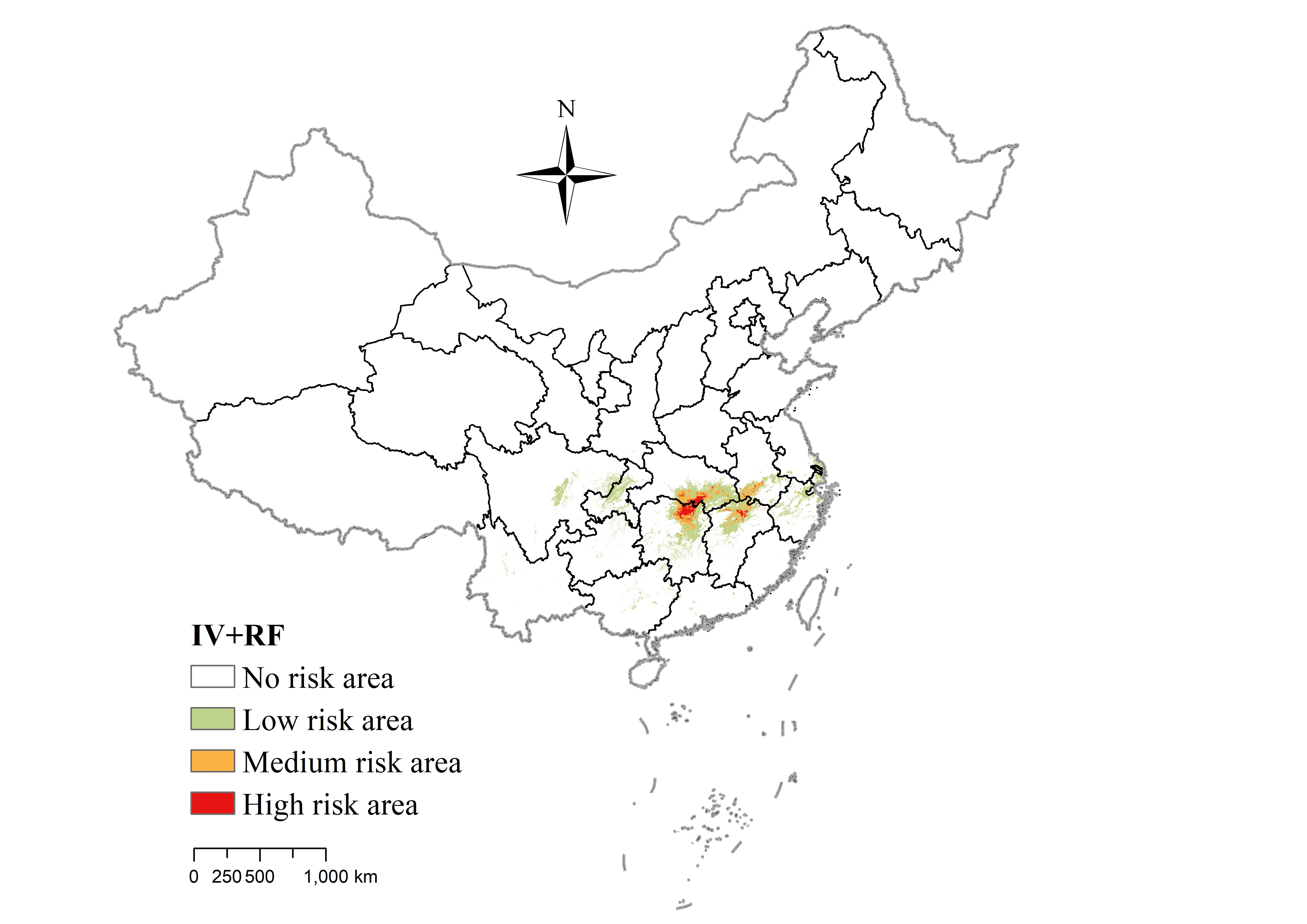

Supplement: Supplementary file 6 — Additional file 6: Figure 6. Current risk prediction for schistosomiasis inChina based on the IV + RF model. [file 40249_2021_874_MOESM6_ESM.jpg]

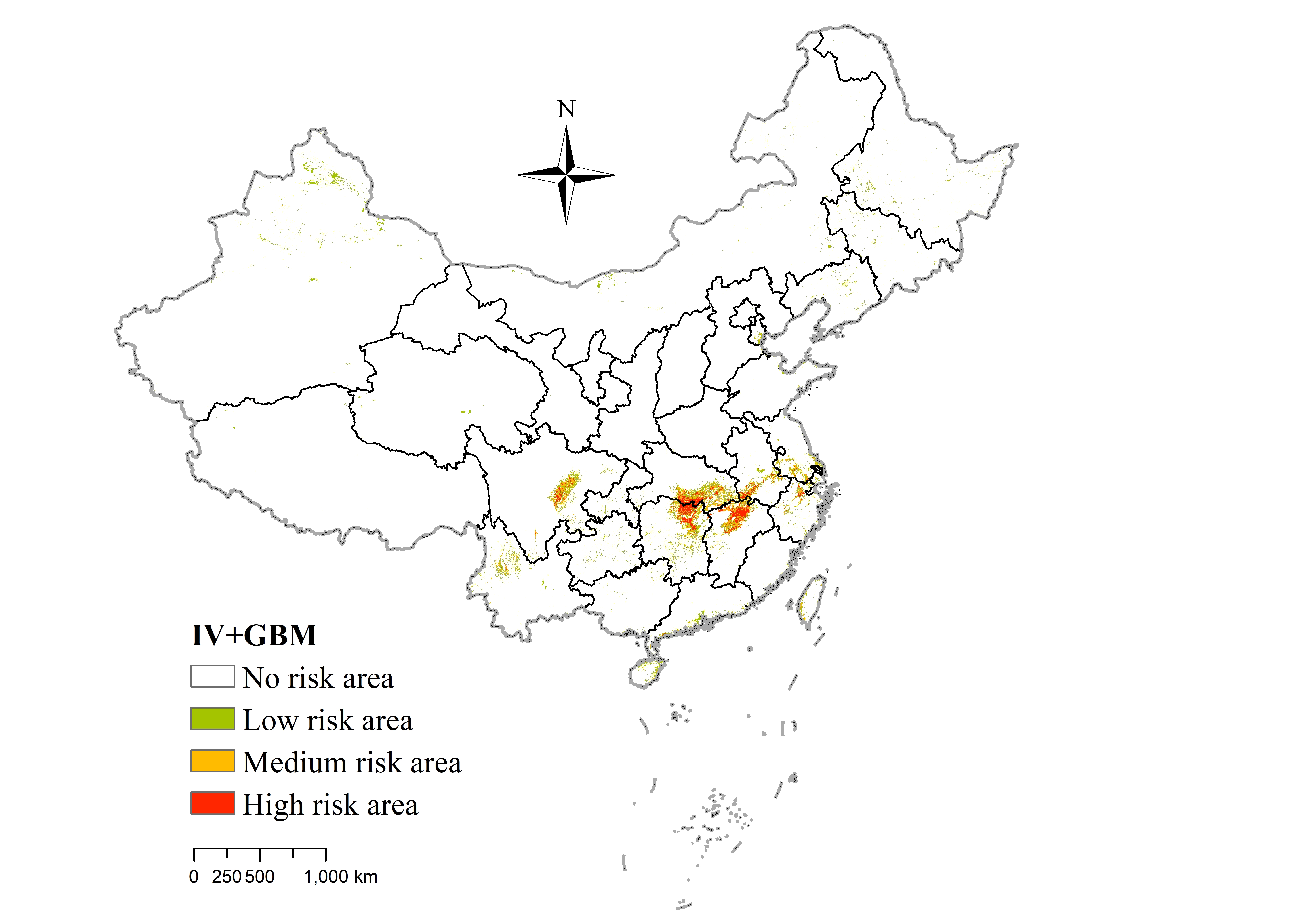

Supplement: Supplementary file 7 — Additional file 7: Figure 7. Current risk prediction for schistosomiasis inChina based on the IV + GBM model. [file 40249_2021_874_MOESM7_ESM.jpg]
